# The Blind Boy

**Published:** 1874-02

**Authors:** Francis L. Hawks


### THE BLIND BOY.

BY REV. FRANCIS L. HAWKS, D.D., LL.D.

It was a blessed summer day,
The flowers bloomed, the air was mild;
The little birds poured forth their lay,
And every thing in nature smiled.

In pleasant thoughts I wandered on
Beneath the deep wood's ample shade,
Till suddenly I came upon
Two children, who had thither strayed.

Just at an aged birch-tree's foot
A little boy and girl reclined—
His hand in hers she kindly put,
And then I saw the boy was blind.

The children knew not I was near—
A tree concealed me from their view;
But all they said I well could hear,
And I could see all they might do.

"Dear Mary," said the poor blind boy,
"That little bird sings very long;
Say, do you see him in his joy?
And is he pretty as his song?"

"Yes, Edward, yes," replied the maid,
"I see that bird on yonder tree."
The poor boy sighed, and gently said:
"Sister, I wish that I could see.

"The flowers, you say, are very fair,
And bright green leaves are on the trees,
And pretty birds are singing there—
How beautiful for one who sees!

"Yet I the fragrant flowers can smell,
And I can feel the green leaf's shade;
And I can hear the notes that swell
From those dear birds that God has made.

"So, sister, God to me is kind,
Though sight, alas! he has not given;
But, tell me, are there any blind
Among the children up in heaven?"

"No, dearest Edward, *there* all see—
But why ask me a thing so odd?"
"O Mary! *he's so good to me,*
*I thought I'd like to look at God.*"

Ere long, disease his hand had laid
On that dear boy, so meek and mild;
His widowed mother wept and prayed
That God would spare her sightless child.

He felt her warm tears on his face,
And said: "Oh! never weep for me;
I'm going to a bright, bright place,
Where, Mary says, *God I shall see.*

"And you'll be there, dear Mary, too;
But, mother, when you get up there,
Tell Edward, mother, that 'tis you—
You know I never saw you here."

He spoke no more, but sweetly smiled
Until the final blow was given,
When God took up that poor blind child,
And opened first his eyes in heaven.

